# PReS-FINAL-2026: Glucose and lipid metabolism in children with juvenile idiopathic arthritis treated with anti-TNF-alpha

**DOI:** 10.1186/1546-0096-11-S2-P39

**Published:** 2013-12-05

**Authors:** AE Christensen, AJ Schou

**Affiliations:** 1 Hans Christian Andersen Childrens Hospital, Odense University Hospital, Odense, Denmark

## Introduction

Inflammation and inflammatory mediators may affect glucose and lipid metabolism through a number of pathways. However, effects and mechanisms seem to vary dramatically in different individuals and settings. In obese adults with metabolic syndrome and impaired glucose-tolerance, inflammatory mediators from fat tissue including tumour necrosis factor-alpha (TNF-α) further reduce glucose tolerance. In such patients, treatment with TNF-α-blocking agents has been shown to ameliorate glucose tolerance. However, in non-obese healthy adults, vigorous exercise may induce an inflammatory response including release of TNF-α. Glucose tolerance increases during and after the exercise, and TNF-α blocking agents may inhibit this beneficial effect. Furthermore, the positive effects of exercise on cholesterol and triglyceride levels have also been diminished by TNF-α blocking agents.

In children with juvenile idiopathic arthritis (JIA) treatment with TNF-α blocking agents is increasingly common as the treatment is efficient and well tolerated. The possible harmful effects of this treatment on glucose and lipid metabolism, however, give rise to concern.

## Objectives

The aim of the study was to investigate the glucose and lipid metabolism in children with JIA treated with TNF-α blocking agents.

## Methods

Fifty-two children with JIA were included in the study. All were treated with immunosuppressive agents: 30 were treated with methotrexate (MTX) in monotherapy, 13 were treated with MTX and a TNF-α blocking agent and 9 were treated with a TNF-α blocking agent in monotherapy. None were treated with oral glucocorticoids. All treatments had been given at least 3 months at the time of blood sampling.

All participants had a fasting blood sample drawn and subsequently the following parameters were assessed: HgbA_1_C (an indicator of the average blood glucose in the 8 weeks prior to testing), fasting blood glucose, total cholesterol, high density lipoprotein (HDL), low density lipoprotein (LDL) and triglyceride.

## Results

Table 1

**Table 1:** 

*Treatment*	*HgbA_1_C/percent*	*Fasting blood glucose/mM*	*Total cholesterol/mM*	*HDL/mM*	*LDL/mM*	*Triglyceride/mM*
*MTX*	5.3 (0.3)	5.0 (0.5)	4.0 (0.7)	1.5 (0.4)	2.1 (0.5)	0.7 (0.3)

*MTX and TNF-α blocker*	5.1 (0.3)	5.0 (0.5)	4.1 (1.2)	1.4 (0.2)	2.3 (0.9)	1,1 (0.8)

*TNF-α blocker*	5.1 (0.2)	4.9 (0.5)	3.9 (0.7)	1.4 (0.3)	2.3 (0.5)	0.6 (0.2)

All values as mean (SD).

The data were tested and found to be normally distributed. Thus, unpaired t-test was used to compare mean-values.

Mean HgbA1C was significantly lower children treated with a TNF-α blocking agent compared to children treated with MTX as monotherapy (p = 0.01). The difference was significant in both children treated with TNF-blocker as monotherapy (p = 0.05) or in combination with MTX (p = 0.03). No other significant differences were detected.

## Conclusion

Children with JIA treated with a TNF-α blocking agent have significantly lower HgbA_1_C. The reduction, however, is small and barely of clinical significance. No effects could be detected on fasting blood glucose or cholesterol levels. The results are reassuring with regard to the concerns for harmful long term side effects of this treatment.

## Disclosure of interest

None declared.

